# Determinants of consumption level among target population in Chinese hot spring medical and long-term care integration with rehabilitation services model and value perception analysis based on the importance-performance analysis framework

**DOI:** 10.3389/fresc.2026.1728803

**Published:** 2026-04-10

**Authors:** Xiaochen Li, Yao Zhou, Zihan Chen, Xue Yang, Liqing Yao

**Affiliations:** 1 Department of Rehabilitation Medicine, The Second Affiliated Hospital of Kunming Medical University, Kunming, China; 2 School of Rehabilitation Medicine, Kunming Medical University, Kunming, China

**Keywords:** consumption behavior, importance-performance analysis (IPA), medical and long-term care integration with rehabilitation services model, medical applications of hot springs, sub-health population

## Abstract

**Objective:**

This cross-sectional empirical study evaluates the Chinese Hot Spring Medical and Long-term Care Integration with Rehabilitation Services Model (Hot Spring MLR Model). By quantifying the demographic characteristics, behavioral determinants, and multidimensional value perceptions of the target population (N=218), this research elucidates developmental bottlenecks and proposes evidence-based optimization strategies.

**Methods:**

Primary empirical data were acquired from target users of the Hot Spring MLR Model (N=218) utilizing a stratified random sampling strategy. The structured instrument evaluated demographic profiles, behavioral determinants, and dual-dimensional Importance-Performance Analysis (IPA) metrics. Predictors of consumption tiers were isolated via univariate screening followed by generalized ordered logistic regression modeling. Furthermore, paired t-tests and IPA matrices were implemented to systematically quantify the divergence between perceived importance and actual service performance.

**Results:**

In the target population survey, the sub-health population constitutes the primary target group for the Hot Spring Medical and Long-term Care Integration with Rehabilitation Services Model (44.5%), with the highest participation rate observed in the traditional Chinese medicine (TCM) of Hot Spring Medical and Long-term Care Integration with Rehabilitation Services Model (82.1%). The consumption behavior within the Hot Spring Medical and Long-term Care Integration with Rehabilitation Services Model is significantly correlated with age, education, consumption motivation, and social media marketing channels (*p* < 0.05). The Importance-Performance Analysis (IPA) of the Hot Spring Medical and Long-term Care Integration with Rehabilitation Services Model reveals a positive correlation between the importance and performance of each sub-dimension. Service attitude demonstrates the highest importance, while professional-quality exhibits the highest performance. Significant discrepancies exist between the importance and performance of the dimensions of affordable pricing and convenient transportation.

**Conclusion:**

Empirical evidence indicates that the optimal service configuration for the Hot Spring MLR Model lies in preventive interventions and holistic health management for sub-health populations. Furthermore, mitigating the quantified behavioral determinants and resolving the supply-demand discrepancies identified via the IPA matrix provide a strictly data-driven framework to optimize resource allocation and enhance overall service efficacy.

## 1 Introduction

The paradigm shift from conventional curative medicine to full-cycle health management in China has exposed critical structural vulnerabilities within the healthcare system (1, 2). The prevailing “prevention absence-rehabilitation delay-nursing disability” trajectory, exacerbated by critical shortages in bed supply and rehabilitation personnel (3–6), manifests as a systemic supply-demand mismatch. Viewed through the lens of the Importance-Performance Analysis (IPA) framework, these institutional and policy frictions translate directly into significant deficits in actual service performance relative to consumer expectations. Consequently, the Hot Spring MLR Model functions not merely as a wellness initiative, but as a strategic, policy-driven mechanism designed to structurally resolve these discrepancies by seamlessly integrating medical interventions, rehabilitation support, and long-term care resources.

China's current health management model also faces significant structural contradictions: medical institutions are limited to disease treatment, but there is a lack of risk prevention for early-stage diseases and inadequate management of chronic diseases. Rehabilitation service institutions simultaneously face a dual imbalance of time and space: delayed intervention post-surgery often leads to missed golden windows for rehabilitation. At the same time, shortages in bed supply and personnel allocation struggle to meet the growing demand from the rehabilitation population. The long-term care system is trapped in a vicious cycle of structural imbalance between supply and demand, deficiencies in integrating medical and older adult care, and inadequate subsequent payment mechanisms. This “prevention absence-rehabilitation delay-nursing disability” chain reflects a systemic disconnect in the current health service system's ability to meet the demands of full-cycle management.

Over the past five years, balneotherapy, as a non-pharmacological and preventative intervention, has garnered significant interest from clinicians and researchers worldwide regarding its application in the medical field (7). As a significant adjunctive modality within rehabilitative physical therapy techniques (8, 9), hydrotherapy utilizing hot springs achieves multifaceted interventions for chronic diseases (10, 11), sub-health conditions (12), and psychological issues (13) through its unique dual action of physical properties and chemical effects (14). Its widespread global application stems from the synergistic benefits of natural components, its environmental healing capacity, a low-risk profile, and lower costs compared to conventional rehabilitation (7). Against this backdrop, the “medical-rehabilitation-older adult care” model, formally known as the Medical and Long-term Care Integration with Rehabilitation Services Model (MLR Model), demonstrates unique value. It achieves seamless integration of disease treatment, functional recovery, and long-term health management by consolidating medical resources, rehabilitation support, and older adult care services. The auxiliary and comprehensive nature of hot spring hydrotherapy, along with its psycho-physiological benefits (15, 16), can be effectively incorporated into the MLR Model. This integration promotes synergistic effects between natural healing and medical interventions while also enabling a more precise alignment of regional natural resources with specific medical needs.

However, as an emerging model within the health industry, the Hot Spring MLR Model currently faces challenges, including an unclear core business positioning, a lack of clarity regarding the behavioral characteristics of the target group, and the absence of a comprehensive value perception and evaluation system. However, a critical research gap persists: while conceptual frameworks for the Hot Spring MLR Model have been extensively proposed, there remains a pronounced deficit of empirical, quantitative investigations elucidating the behavioral determinants and multidimensional value perceptions of its target demographic. To directly address this empirical void, the present cross-sectional study leverages primary survey data to systematically delineate the demographic profiles, thereby establishing a robust, data-driven foundation for the model's clinical and commercial application within the Chinese context. A multivariate logistic regression analysis is conducted to determine the key factors associated with the consumption behavior of the target population.

To systematically address this empirical void, the present research is theoretically anchored in the Importance-Performance Analysis (IPA) framework (17). Serving as the core analytical lens, the IPA paradigm provides a robust quantitative mechanism for evaluating the discrepancy between consumer expectations and actual service delivery. By integrating demographic and behavioral determinants into this multidimensional framework, we mathematically map the structural supply-demand mismatches within the Hot Spring MLR Model. Guided by this conceptual framework, the subsequent sections systematically unfold the empirical design: we first detail the stratified sampling methodology and target population profiles, subsequently map the behavioral predictors and value discrepancies via quantitative modeling, and ultimately synthesize these findings into data-driven policy implications for the industry.

## 2 Materials and methods

### 2.1 Research subjects

This study, conducted in collaboration with the China Hot Spring Association, focuses on domestic Hot Spring MLR Model institutions and employs a cross-sectional survey methodology. This study employed a stratified random sampling method to distribute 240 standardized questionnaires titled “Target Population Survey of Hot Spring MLR Model Institutions” across the country. A total of 218 valid responses were collected, resulting in an effective response rate of 90.83%. The study defined valid questionnaires as those with no more than five missing data items or exhibiting no patterned response tendencies.

This cross-sectional survey study, conducted in collaboration with the China Hot Spring Association, focuses on domestic Hot Spring MLR Model institutions. The researchers employed a stratified random sampling approach to administer 240 standardized questionnaires entitled “Target Population Survey of Hot Spring MLR Model Institutions” across the country. A total of 218 valid responses were collected, yielding an effective response rate of 90.83%. Questionnaires were considered valid after excluding those with more than five missing items or showing patterned response tendencies.

The inclusion criteria encompass both institutional qualifications and respondent eligibility.

1. Institutional Qualification Criteria:
   ① Holding a valid hot spring development license and health safety certification;
   ② Having continuously operated hot spring wellness programs for at least three years;
   ③ Being staffed with full-time health management professionals or rehabilitation medicine specialists.
2. Respondent Eligibility Criteria:
   ① Being familiar with the organization's operations and capable of completing the questionnaire independently or with assistance;
   ② Belonging to the target population served by Chinese Hot Spring MLR Model institutions that specialize in hot spring therapy.

### 2.2 Ethical declaration

The study obtained ethical approval from the Ethics Committee of The Second Affiliated Hospital, Kunming Medical University (Reference No. FEY-BG-39-2.0). All participants gave their informed consent and voluntarily participated in the study.

### 2.3 Research methodology

This study, conducted in July 2024, involved the development of the Target Population Survey of Hot Spring MLR Model Institutions following a systematic literature review. The study employed a cross-sectional design and was administered online via the Wenjuanxing (Questionnaire Star) platform, targeting institutions in China that use the Hot Spring MLR Model. Non-probability convenience sampling was conducted based on geographic distribution characteristics. The target population was limited to individuals who were able to operate electronic devices independently and willing to participate in the study, with a specific focus on users of Chinese Hot Spring MLR Model services.

### 2.4 Research tools

Anchored in the strategic directives established by the State Council's Guiding Opinions on Promoting the Integration of Medical and Healthcare Services with older adult Care (18), the instrument development process utilized a rigorous, multi-stage methodology.

To ensure rigorous content validity, the initial generation of survey items was predicated on a targeted literature retrieval strategy rather than relying on an unstructured literature review. Specifically, comprehensive database querying was executed across PubMed, Web of Science, and the China National Knowledge Infrastructure (CNKI), with the temporal parameter strictly limited to peer-reviewed publications between 2014 and 2024. The inclusion criteria for source selection mandated that studies specifically evaluate hot spring balneotherapy mechanisms, integrated rehabilitation frameworks, or quantitative consumer value perceptions. Theoretical constructs extracted from this rigorously selected literature directly operationalized the dimensions within our Importance-Performance Analysis (IPA) matrix. Following this data-driven item generation, the draft instrument, titled “Target Population Survey of Hot Spring MLR Model Institutions,” underwent structural refinement via an expert panel validation process.

To empirically contextualize these theoretical dimensions, semi-structured qualitative interviews (N=10) were subsequently conducted with representative target users at the Health Rehabilitation Center of Yunnan Province and the Tengchong East Lake Hot Spring Rehabilitation Medical Center. Psychometric evaluation of the finalized instrument, titled “Target Population Survey of Hot Spring MLR Model Institutions,” confirmed exceptional internal consistency, yielding a Cronbach's α of 0.948.

### 2.5 Quality control

In July 2024, The Second Affiliated Hospital of Kunming Medical University, Wenzhou Medical University, and the China Hot Springs Association jointly established a research task force named Hot Spring MLR Model Investigation to conduct an in-depth study of the model.

Before formally launching the study, the research implementation team conducted standardized training. The research team designed this training to ensure the consistent application of guidelines when explaining research objectives and data collection procedures to participants, thereby cultivating respondent trust and cooperation. During the survey process, field staff distributed and collected all questionnaires independently and anonymously. This approach prevented privacy intrusion and maintained the authenticity of the response. For data entry, verification personnel implemented rigorous double-entry verification and cross-checking procedures to ensure data accuracy and reliability.

### 2.6 Preliminary experiment

Prior to the formal survey, considering heterogeneity factors such as geographic location and scale, two representative Hot Spring MLR Model institutions were selected in Yunnan, a region rich in hot spring resources in China, representing different levels and types. These institutions were the Yunnan Province Health Rehabilitation Center and the Tengchong East Lake Hot Spring Rehabilitation Medical Center. The research team conducted a pilot survey with ten participants involved in the Hot Spring MLR Model. Based on the pilot survey results, the questionnaire content was revised and adjusted through modifications, deletions, and restructuring to ensure participants could better understand the questions and answer them independently.

### 2.7 Statistical analysis

Data entry was independently performed by two trained personnel using EpiData 3.1 software, with consistency checks implemented to ensure data quality and accuracy. We conducted statistical analyses using SPSS 22.0 and Stata 17.0. We used descriptive statistics to summarize the demographic and consumer behavior characteristics of the target population for the Chinese Hot Spring Multiple Linear Regression (MLR) Model. We assessed the normality of continuous variables using the Shapiro–Wilk test. Variables conforming to a normal distribution were expressed as mea*n* ± standard deviation (x¯ ± s), and we performed group comparisons using independent samples t-tests. We described categorical variables using frequencies and percentages [n (%)], and we analyzed differences between groups using Pearson's *χ*^2^ test. When expected cell counts were less than 5, continuity correction or Fisher's exact test was applied.

We performed preliminary screening of potential predictor variables using univariate analysis (significance threshold *p* < 0.05). Subsequently, we initially fitted an ordinal logistic regression model to identify factors significantly associated with consumption levels. However, the Brant test indicated a violation of the proportional odds assumption (*χ*^2^ = 490.906, *p* < 0.001). We, therefore, implemented Williams' generalized ordered logit model (estimated via Stata's gologit2 command) for parameter estimation. This flexible approach simultaneously accommodates variables that meet the proportional odds assumption and variables that demonstrate category-specific effects. The ordinal dependent variable, “consumption level,” consisted of six categories, ranging from 1 (lowest) to 6 (highest). Model construction generated five distinct cumulative logit functions: Levels 2–6 vs. Level 1 (reference); Levels 3–6 vs. Levels 1–2; Levels 4–6 vs. Levels 1–3; Levels 5–6 vs. Levels 1–4; Level 6 vs. Levels 1–5. Additionally, we assessed interrelationships among perceived value dimensions within the Chinese Hot Spring MLR Model using correlation analysis. Paired samples t-tests evaluated the magnitude of value perception differences. We employed Importance-Performance Analysis (IPA) to assess the significance and performance of each variable within the IPA matrix (19): Quadrant I (Keep Up the Good Work): High Importance, High Performance; Quadrant II (Concentrate Here): High Importance, Low Performance; Quadrant III (Low Priority): Low Importance, Low PerformanceQuadrant IV (Possible Overkill): Low Importance, High Performance. We conducted all statistical analyses using two-sided tests with an *α* = 0.05 significance threshold. We report effect sizes with 95% confidence intervals (95% CI).

## 3 Result

### 3.1 Participant characteristics and single-factor analysis of consumption levels

**Figure 1:**
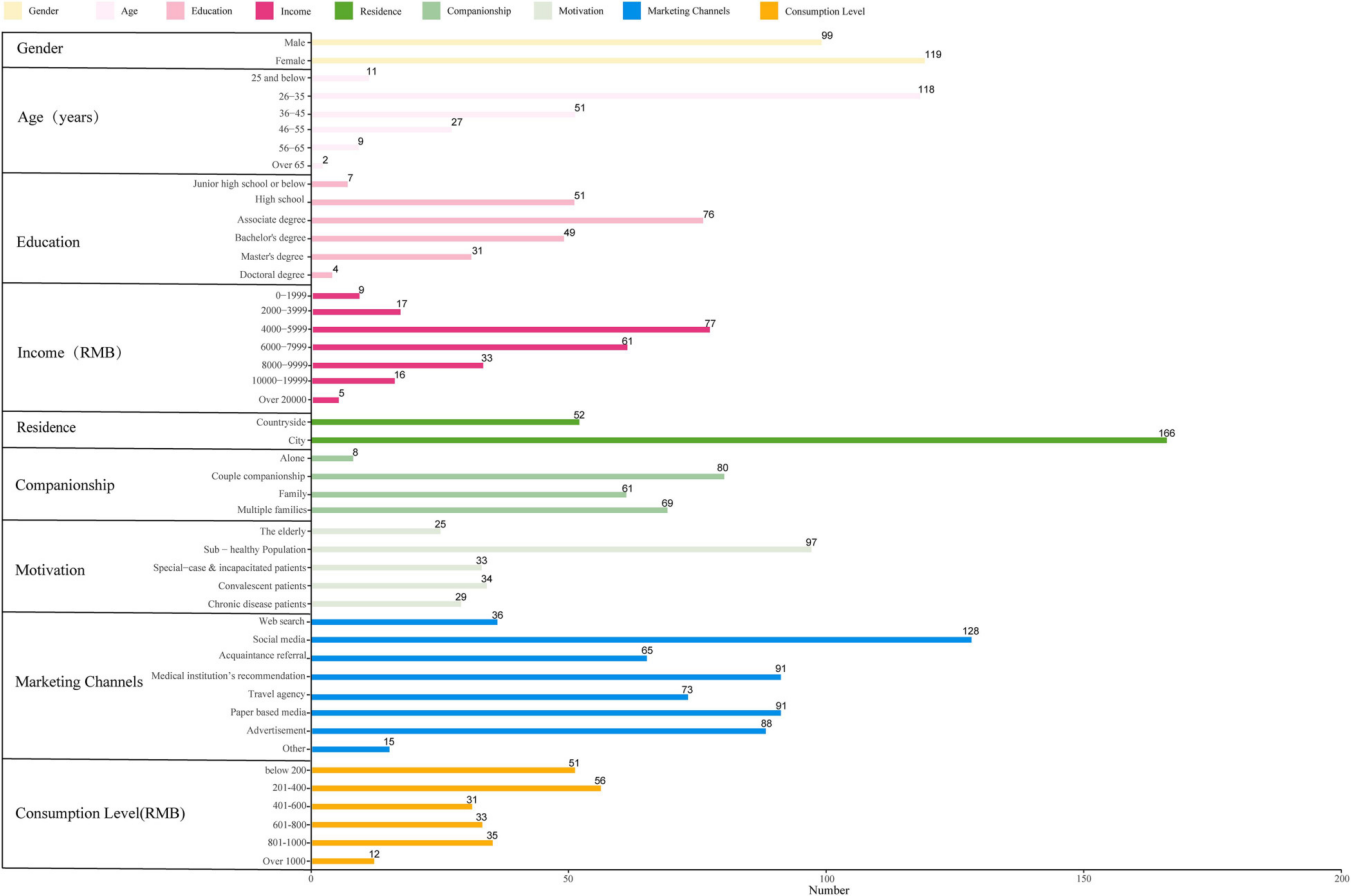
Demographic and behavioral profiles of the target population within the Chinese Hot spring MLR model. Plotted by the authors utilizing primary cross-sectional survey data (N=218). Horizontal bars represent the absolute frequency (count) of respondents across distinct demographic, socioeconomic, and behavioral categories.

This study surveyed 218 participants from the target population. Figure 1 details the socio-demographic characteristics of individuals engaging with the Chinese Hot Spring MLR Model. Chi-square tests revealed significant differences in consumption levels across age groups, educational attainment, consumption motivation, and social media promotional channels (*p* < 0.05; Table 1).

**Table 1:** The relationship between the general circumstances of target groups and their purchase behavior.

		Consumption Level (RMB)							
Items	Number	Below 200	200–399	400–599	600–799	800–999	Over 1,000	*χ*2 value	*P* value
Gender								4.015	0.547
Male	99	25（25.3%）	27（27.3%）	14（14.1%）	15（15.2%）	11（11.1%）	7（7.1%）		
Female	119	26（21.8%）	29（24.4%）	17（14.3%）	18（15.1%）	24（20.2%）	5（4.2%）		
Age								42.095	0.018*
25 and below	11	5（45.5%）	6（54.5%）	0	0	0	0		
26–35	118	33（28.0%）	19（16.1%）	20（16.9%）	20（16.9%）	18（15.3%）	8（6.8%）		
36–45	51	7（13.7%）	19（37.3%）	5（9.8%）	10（19.6%）	7（13.7%）	3（5.9%）		
46–55	27	4（14.8%）	6（22.2%）	4（14.8%）	2（7.4%）	10（37.0%）	1（3.7%）		
56–65	9	1（11.1%）	5（55.6%）	2（22.2%）	1（11.1%）	0	0		
over 65	2	1（50%）	1（50%）	0	0	0	0		
Education								41.188	0.022*
Junior high school or below	7	1（14.3%）	2（28.6%）	3（42.9%）	0	0	1（14.3%）		
High school	51	15（29.4%）	11（21.6%）	2（3.9%）	4（7.8%）	14（27.5%）	5（9.8%）		
Associate degree	76	21（27.6%）	20（26.3%）	10（13.2%）	16（21.1%）	9（11.8%）	0		
Bachelor's degree	49	9（18.4%）	14（28.6%）	8（16.3%）	8（16.3%）	4（8.2%）	6（12.2%）		
Master's degree	31	4（12.9%）	8（25.8%）	7（22.6%）	5（16.1%）	7（22.6%）	0		
Doctoral degree	4	1（25%）	1（25%）	1（25%）	0	1（25%）	0		
Income								39.632	0.112
0–1,999	9	5（55.6%）	4（44.4%）	0	0	0	0		
2,000–3,999	17	3（17.6%）	4（23.5%）	4（23.5%）	4（23.5%）	2（11.8%）	0		
4,000–5,999	77	21（27.3%）	21（27.3%）	7（9.1%）	10（13.0%）	12（15.6%）	6（7.8%）		
6,000–7,999	61	12（19.7%）	11（18.0%）	11（18.0%）	11（18.0%）	12（19.7%）	4（6.6%）		
8,000–9,999	33	4（12.1%）	15（45.5%）	4（12.1%）	4（12.1%）	4（12.1%）	2（6.1%）		
10,000–19,999	16	6（37.5%）	4（25.0%）	3（18.8%）	2（12.5%）	4（25.0%）	0		
Over 20,000	5	0	0	2（40.0%）	2（40.0%）	1（20.0%）	0		
Residence								4.650	0.46
Countryside	52	12（23.1%）	16（30.8%）	6（11.5%）	4（7.7%）	11（21.2%）	3（5.8%）		
City	166	39（23.5%）	40（24.1%）	25（15.1%）	29（17.5%）	24（14.5%）	9（5.4%）		
Companionship								15.897	0.389
Alone	8	3（37.5%）	2（25%）	0	3（37.5%）	0	0		
Couple companionship	80	17（21.3%）	20（25%）	12（15%）	10（12.5%）	15（18.8%）	6（7.5%）		
Family	61	14（23.0%）	15（24.6%）	8（13.1%）	7（11.5%）	15（24.6%）	2（3.3%）		
Multiple families	69	17（24.6%）	19（27.5%）	11（15.9%）	13（18.8%）	5（7.2%）	4（5.8%）		
Motivation								47.627	<0.001***
The older adult	25	5（20.0%）	4（16.0%）	5（20.0%）	5（20.0%）	6（24.0%）	0		
Sub−healthy Population	97	18（18.6%）	20（20.6%）	11（11.3%）	19（19.6%）	20（20.6%）	9（9.3%）		
Special−case & incapacitated patients	33	9（27.3%）	14（14.4%）	2（6.1%）	2（6.1%）	5（15.2%）	1（3.0%）		
Convalescent patients	34	6（17.6%）	7（20.6%）	11（32.4%）	7（20.6%）	3（8.8%）	0		
Chronic disease patients	29	13（44.8%）	11（37.9%）	2（6.9%）	1（3.4%）	2（6.9%）	0		
Marketing Channels									
Web search	36	7（19.4%）	9（25.0%）	9（25.0%）	3（8.3%）	6（16.7%）	2（5.6%）	5.148	0.398
Social media	128	20（15.6%）	28（21.9%）	22（17.2%）	23（18.0%）	24（18.8%）	11（8.6%）	20.094	0.001**
Acquaintance referral	65	18（27.7%）	16（24.6%）	4（6.2%）	14（21.5%）	11（16.9%）	2（3.1%）	8.552	0.128
Medical institution's recommendation	91	23（25.3%）	16（17.6%）	13（14.3%）	16（17.6%）	19（20.9%）	4（4.4%）	7.462	0.189
Travel agency	73	17（23.3%）	23（31.5%）	11（15.1%）	5（6.8%）	11（15.1%）	6（8.2%）	8.019	0.155
Paper based media	91	19（20.9%）	27（29.7%）	11（12.1%）	12（13.2%）	18（19.8%）	4（4.4%）	3.978	0.553
Advertisement	88	22（25.0%）	22（25.0%）	8（9.1%）	14（15.9%）	16（18.2%）	6（6.8%）	3.856	0.570
Other	15	2（13.3%）	5（33.3%）	3（20.0%）	3（20.0%）	0	2（13.3%）	6.073	0.299

Computed by the authors utilizing primary empirical survey data (N=218). The table presents cross-tabulations and Pearson's Chi-square ($\chi^{2}$) tests evaluating the association between target demographic/behavioral characteristics and corresponding consumption levels. Data are presented as absolute frequencies with row percentages in parentheses. Statistical significance is denoted as follows: * P<0.05, ** P<0.01, *** P<0.001.

### 3.2 Trends in the Chinese hot spring MLR model consumption

The Traditional Chinese Medicine Type demonstrated the highest participation rate (82.1%, *n* = 179) among all the Chinese Hot Spring MLR Models analyzed (Table 2).

**Table 2:** The participation status of target population in all types of Hot spring medical and long-term care integration with rehabilitation services model activities.

Classification	Number of participants	Below 200	201–400	401–600	601–800	801–1,000	Over 1,000
Western Medicine Type	132（60.6%）	27（20.5%）	35（26.5%）	11（8.3%）	23（17.4%）	24（18.2%）	12（9.1%）
Traditional Chinese Medicine Type	179（82.1%）	39（21.8%）	43（24.0%）	28（15.6%）	29（16.2%）	31（17.3%）	9（5.0%）
Non-Medical Type	160（73.4%）	37（23.1%）	41（25.6%）	23（14.4%）	28（17.5%）	29（18.1%）	6（3.8%）
Other	55（25.2%）	10（18.2%）	10（18.2%）	13（23.6%）	3（5.5%）	14（25.5%）	5（9.1%）

Data compiled and synthesized by the authors utilizing primary cross-sectional survey data (N=218). Data are presented as absolute frequencies (counts) with corresponding percentages in parentheses. Note that percentages in the “Number of participants” column represent the proportion relative to the total study sample (N=218), whereas percentages in the subsequent consumption level categories represent row percentages within each specific activity type.

### 3.3 Determinants of target population spending on the Chinese hot spring MLR model

Generalized ordered logistic regression analysis identified key determinants of Chinese Hot Spring MLR Model expenditure levels (Table 3). Age demonstrated a significant positive association with expenditure, indicating that older participants spent more. Motivations and social media promotional channels showed significant inverse associations. Educational attainment exhibited differential effects: positive among high school graduates but negative among doctoral degree holders (all associations *p* < 0.05).

**Table 3:** Generalized ordered logistic regression analysis of determinants associated with expenditure tiers among target groups in the Hot spring medical and long-term care integration with rehabilitation services model.

Variables	Model 1: Levels 2–6 vs. Level 1 (reference)			Model 2: Levels 3–6 vs. Levels 1–2			Model 3: Levels 4–6 vs. Levels 1–3			Model 4: Levels 5–6 vs. Levels 1–4			Model 5: Levels 6 vs. Levels 1–5		
	*β*	95% CI	z	β	95% CI	z	β	95% CI	z	β	95% CI	z	β	95% CI	z
Age (ref:25 and below)															
26–35	0.67	−0.63,1.98	1.01	2.52	1.05,3.99	3.37**	1.21	−0.42,2.84	1.46	1.76	0.08,3.44	2.05*	1.19	−1.26,3.65	0.95
36–45	1.72	0.32,3.12	2.40*	1.72	0.32,3.12	2.40*	1.72	0.32,3.12	2.40*	1.72	0.32,3.12	2.40*	1.72	0.32, 3.12	2.40*
46–55	1.94	0.28,3.60	2.30*	1.94	0.28,3.60	2.30*	1.94	0.28,3.60	2.30*	1.94	0.28,3.60	2.30*	1.94	0.28, 3.60	2.30*
56–65	1.74	−0.80,4.28	1.34	−0.16	−2.58,2.25	−0.13	−0.80	−3.63,2.03	−0.55	−2.04	−7.60,3.52	−0.72	5.00	2.77, 7.23	4.39***
Over 65	−1.73	−5.15,1.69	−0.99	−1.73	−5.15,1.69	−0.99	−1.73	−5.15,1.69	−0.99	−1.73	−5.15,1.69	−0.99	−1.73	−5.15,1.69	−0.99
Education (ref:Junior high school or below)															
High school	−1.05	−2.74,0.65	−1.21	−0.39	−2.06,1.27	−0.46	2.05	0.35,3.75	2.37*	2.14	0.40,3.88	2.41*	0.26	−1.96,2.47	0.23
Associate degree	−0.45	−2.08,1.19	−0.54	0.36	−1.16,1.89	0.47	0.40	−1.16,1.96	0.50	−0.42	−2.11,1.28	−0.48	−5.29E + 12		
Bachelor's degree	0.07	−1.48,1.61	0.08	0.07	−1.48,1.61	0.08	0.07	−1.48,1.61	0.08	0.07	−1.48,1.61	0.08	0.07	−1.48,1.61	0.08
Master's degree	0.17	−1.74,2.07	0.17	1.05	−0.66,2.75	1.21	−0.28	−2.01,1.44	−0.32	0.19	−1.65,2.03	0.20	−21.98		
Doctoral degree	−3.26	−6.47,-0.06	-2.00*	-1.85	-4.88,1.19	-1.19	-1.39	-6.32,3.54	-0.55	6.41	-7.25,20.07	0.92	-19.27		
Motivation (ref: The older adult)															
Sub−healthy Population	-0.52	-2.23,1.19	-0.59	-4.34	-6.56,-2.11	-3.82***	-6.92	-12.62,-1.21	-2.38*	3.14	-4.52,10.80	0.80	24.39		
Special−case & incapacitated patients	0.68	-0.96,2.32	0.81	-1.73	-3.38,-0.09	-2.07*	0.08	-1.33,1.49	0.11	-0.16	-1.63,1.31	-0.21	3.50	-3.07,10.07	1.04
Convalescent patients	1.69	-0.14,3.52	1.81	-0.87	-2.65,0.91	-0.96	-1.21	-2.83,0.41	-1.46	-2.01	-3.84,-0.18	-2.15*	-31.21		
Chronic disease patients	0.15	-1.47,1.77	0.18	-2.68	-4.34,-1.03	-3.18**	-2.09	-3.67,-0.51	-2.59*	-1.35	-3.04,0.34	-1.56	2.57	-4.18,9.33	0.75
Marketing Channels (ref: Non-social media)															
Social media	-0.88	-1.46,-0.29	-2.95*	-0.88	-1.46,-0.29	-2.95**	-0.88	-1.46,-0.29	-2.95**	-0.88	-1.46,-0.29	-2.95**	-0.88	-1.46,-0.29	-2.95**
Constant	0.52	-1.97,3.02	0.41	-0.23	-2.68,2.22	-0.18	-1.22	-3.64,1.20	-0.99	-2.13	-4.63,0.38	-1.66	-6.03	-12.75,0.69	-1.76

Computed by the authors utilizing primary empirical survey data (N=218). The table presents unstandardized coefficients (β), 95% confidence intervals (95% CI), and z -statistics derived from the generalized ordered logistic regression models. Statistical significance is denoted as follows: * P<0.05, ** P<0.01, *** P<0.001.

### 3.4 Analysis of the Chinese hot spring MLR model based on the IPA framework

#### 3.4.1 Descriptive analysis

Descriptive analysis of the Chinese Hot Spring MLR Model sub-dimensions (Table 4) revealed acceptable normality parameters: skewness values ranged from 0.428 to 0.800 and kurtosis from 0.013 to 0.404, within established thresholds (±3.0 for skewness; ±10.0 for kurtosis) (20, 21). Service attitude emerged as the most critical component of the model experience.

**Table 4:** Importance-performance analysis of descriptive statistics.

Variables		Means	Rank	Standard Error	Skewness	Kurtosis
Importance	Affordable Price	3.51	5	1.096	−0.568	−0.257
	Business Model	3.68	2	1.119	−0.435	−0.277
	Convenient Transportation	3.50	6	1.040	−0.800	0.082
	Hot spring Culture	3.54	4	1.091	−0.428	−0.336
	Professional Quality	3.56	3	1.069	−0.472	−0.261
	Service Attitude	3.77	1	1.101	−0.675	−0.153
Performance	Affordable Price	3.47	6	1.065	−0.793	0.013
	Business Model	3.54	5	1.164	−0.656	−0.359
	Convenient Transportation	3.62	3	1.163	−0.607	−0.350
	Hot spring Culture	3.71	2	1.239	−0.735	0.103
	Professional Quality	3.77	1	1.112	−0.760	−0.404
	Service Attitude	3.55	4	1.124	−0.531	−0.347

Data compiled and synthesized by the authors utilizing primary empirical survey data (N=218). All service dimensions were evaluated using a standard 5-point Likert scale. “Rank” denotes the ordinal prioritization based on mean scores within the respective Importance and Performance domains. Skewness and kurtosis indices are reported to verify the distributional normality of the empirical sample.

#### 3.4.2 Correlation analysis

Correlation analysis revealed significant positive associations between the importance and performance of all Chinese Hot Spring MLR Model sub-dimensions (Tables 5, 6). Correlation coefficients ranged from 0.472 to 0.711 for importance and 0.343 to 0.648 for performance. No correlations reached the threshold for strong relationships (r > 0.800).

**Table 5:** Correlation analysis of the Chinese Hot spring MLR model importance.

Variables	Affordable price	Business model	Convenient transportation	Hot spring culture	Professional quality	Service attitude
A	1.000					
B	0.460***	1.000				
C	0.616***	0.450***	1.000			
D	0.420***	0.589***	0.455***	1.000		
E	0.397***	0.587***	0.371***	0.468***	1.000	
F	0.466***	0.576***	0.455***	0.534***	0.506***	1.000

Bivariate correlation matrix computed by the authors utilizing primary empirical survey data (N=218). Variable designations: A = Affordable Price; B = Business Model; C = Convenient Transportation; D = Hot spring Culture; E = Professional Quality; F = Service Attitude. Statistical significance (two-tailed) is denoted as follows: *** P<0.001.

**Table 6:** Correlation analysis of the Chinese Hot spring MLR model performance.

Variables	Affordable price	Business model	Convenient transportation	Hot spring culture	Professional quality	Service attitude
A	1.000					
B	0.609***	1.000				
C	0.432***	0.424***	1.000			
D	0.461***	0.399***	0.496***	1.000		
E	0.583***	0.543***	0.490***	0.455***	1.000	
F	0.441***	0.403***	0.469***	0.541***	0.404***	1.000

Bivariate correlation matrix computed by the authors utilizing primary empirical survey data (N=218). Variable designations: A = Affordable Price; B = Business Model; C = Convenient Transportation; D = Hot spring Culture; E = Professional Quality; F = Service Attitude. Statistical significance (two-tailed) is denoted as follows: *** Plt0.001.

#### 3.4.3 Analysis of the importance and performance variations of dimensions

Paired samples *t* tests revealed significant differences between importance and performance ratings for affordable price and convenient transportation dimensions (Table 7). Convenient transportation demonstrated the most considerable performance-importance discrepancy among all dimensions.

**Table 7:** Analysis of the difference between importance and performance by categories.

Sub-Dimensions	Importance		Performance		Mean Difference	Rank	t
	Mean	Standard Deviation	Mean	Standard Deviation			
Affordable Price	3.51	1.096	3.47	1.065	−0.216	2	−2.759*
Business Model	3.68	1.119	3.54	1.164	−0.115	5	−1.407
Convenient Transportation	3.5	1.04	3.62	1.163	0.220	1	2.855*
Hot spring Culture	3.54	1.091	3.71	1.239	0.041	6	0.548
Professional Quality	3.56	1.069	3.77	1.112	−0.165	3	−1.872
Service Attitude	3.77	1.101	3.55	1.124	0.142	4	1.766

Computed by the authors utilizing primary empirical survey data (N=218). Paired-samples t-tests were executed to evaluate the mean discrepancies between perceived consumer expectations (Importance) and actual service delivery (Performance) across the six service dimensions. All items were assessed using a standard 5-point Likert scale. Statistical significance (two-tailed) is denoted as follows: * P<0.05.

#### 3.4.4 Importance-performance analysis matrix

**Figure 2:**
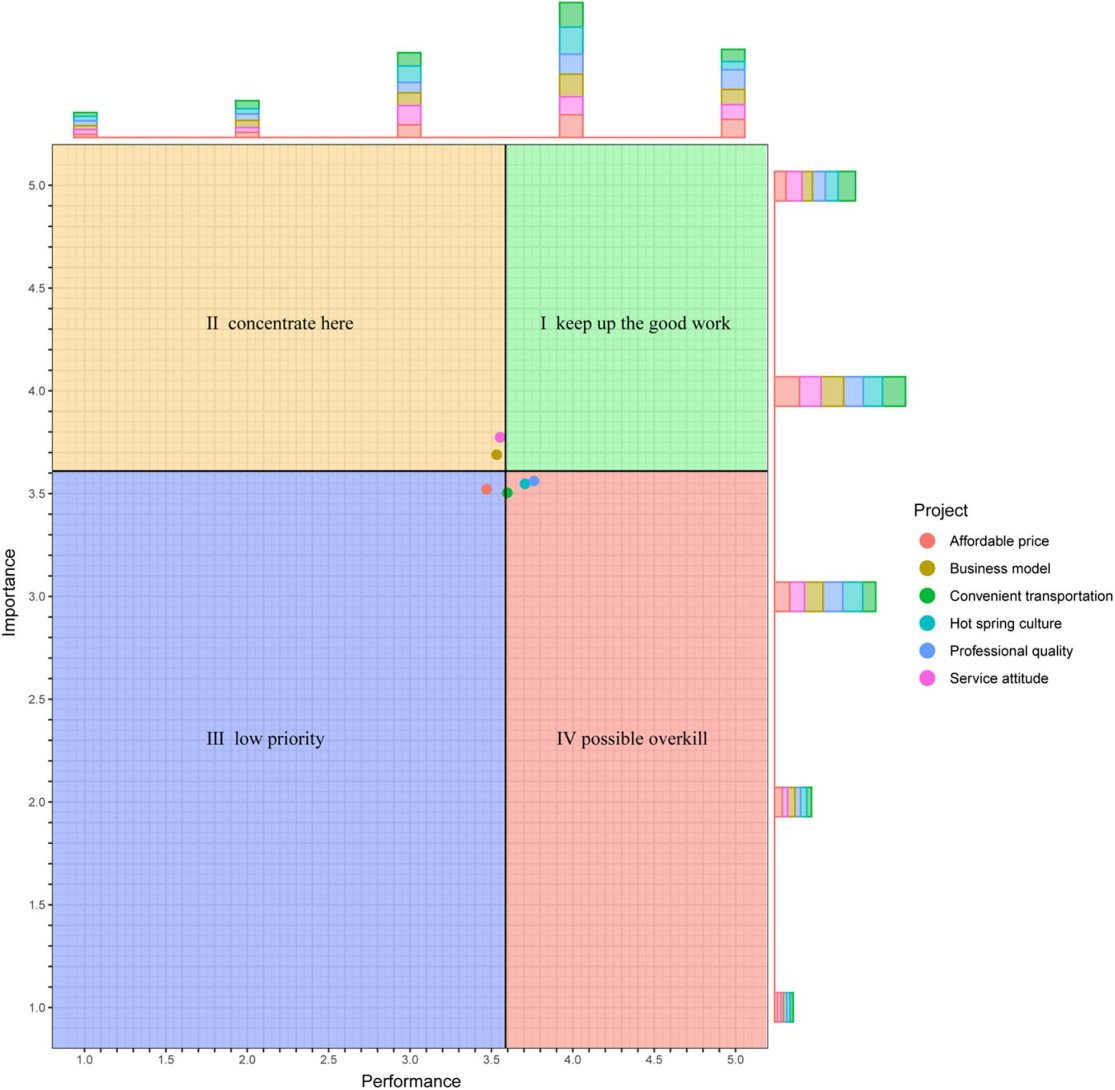
Importance-Performance analysis (IPA) quadrant distribution of the Chinese Hot spring MLR model. Plotted by the authors utilizing quantitative Importance-Performance Evaluation metrics derived from the primary target population cohort (N=218). The matrix evaluates the relationship between perceived project importance and actual performance ratings. The four colored quadrants are defined based on the grand means of the importance and performance scores across all six dimensions. The quadrants correspond to the standard IPA classification framework: Green for keep up the good work (high importance, high performance), Tan for concentrate here (high importance, low performance), Blue for low priority (low importance, low performance), and Red for possible overkill (low importance, high performance). Colored dots represent the grand mean position of six specific project attributes (e.g., Affordable price, Business model), which can be identified by referring to the accompanying “Project” legend. The stacked bar plots on the top and right margins display response distributions for each project dimension, complementing the dot-matrix representation of grand means.

We constructed the IPA matrix using median importance (3.59) and performance scores (3.61) as quadrant thresholds (19) (Figure 2). This analytical approach assessed the strategic positioning of all dimensions within China's hot spring medical wellness model. Notably, Table 8 reveals that no dimensions currently occupy Quadrant I (high importance/high performance).

**Table 8:** Distribution of dimensions of the Chinese Hot spring MLR model.

Quadrant	Characteristics	Dimensions
Quadrant Ⅰ	Importance↑, performance↑	None
Quadrant Ⅱ	Importance↑, performance↓	Business Model,Service Attitude
Quadrant Ⅲ	Importance↓, performance↓	Affordable Price
Quadrant Ⅳ	Importance↓, performance↑	Hot spring Culture,Professional Quality,Convenient Transportation

Dimensional distribution compiled by the authors utilizing primary empirical survey data (N=218). The classification into the Importance-Performance Analysis (IPA) quadrants is predicated on the grand means of the importance and performance scores across all dimensions. Upward arrows (↑) designate mean scores positioned above the grand mean (High), whereas downward arrows (↓) designate mean scores positioned below the grand mean (Low).

### 3.5 Analysis of common types of the Chinese hot spring MLR model

#### 3.5.1 Descriptive analysis of various dimensions of the typical Chinese hot spring MLR model

**Figure 3:**
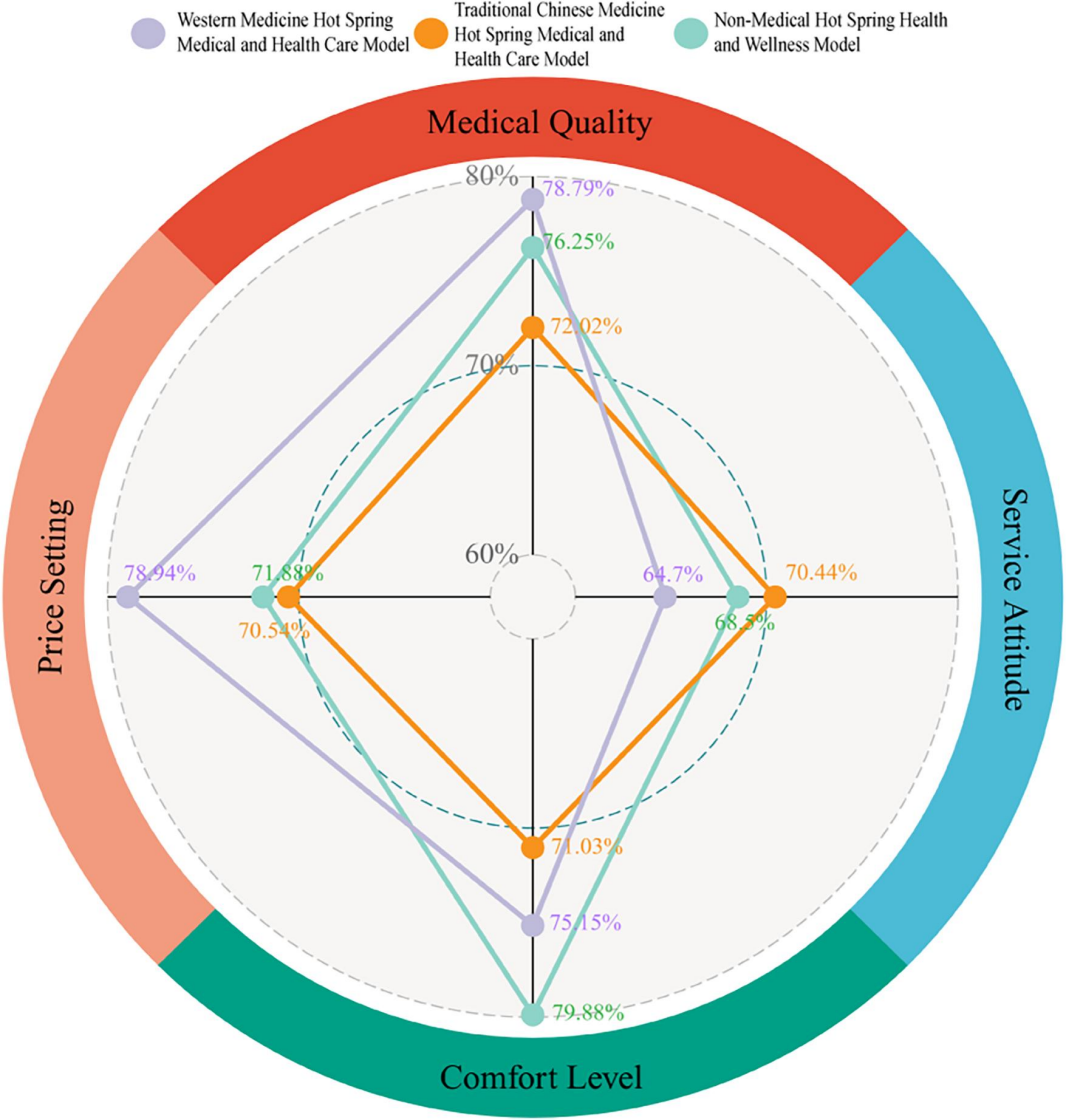
Multidimensional comparative evaluation of the Chinese Hot spring MLR operational typologies. Plotted by the authors utilizing primary empirical survey data (N=218). The radar chart presents a multidimensional performance comparison across three distinct operational models: Western Medicine (purple line), Traditional Chinese Medicine (orange line), and Non-Medical (green line). The four vertices of the radar chart represent the core evaluative dimensions: Medical Quality, Service Attitude, Comfort Level, and Price Setting. Data points depict standardized percentage scores derived from consumer evaluations, facilitating a direct cross-sectional comparison of relative operational strengths and service configurations among the specific typologies.

Descriptive analysis reveals distinguishing characteristics across Chinese Hot Spring MLR Model types (Table 9, Figure 3): the Western Medicine Type features prominent medical quality and pricing settings. Traditional Chinese Medicine Type prioritizes service attitude (highest scoring dimension). Non-medical Type emphasizes comfort level as their defining attribute.

**Table 9:** Multidimensional descriptive analysis of various types of the Chinese Hot spring MLR model.

Classification	Dimensions	Mean	Standard Error
Western Medicine Type	Medical Quality	3.94	1.358
	Service Attitude	3.23	1.235
	Comfort Level	3.76	1.303
	Price Setting	3.95	1.315
Traditional Chinese Medicine Type	Medical Quality	3.60	1.021
	Service Attitude	3.52	1.145
	Comfort Level	3.55	1.152
	Price Setting	3.53	1.105
Non-Medical Type	Medical Quality	3.81	1.260
	Service Attitude	3.42	1.247
	Comfort Level	3.99	1.073
	Price Setting	3.59	1.173

Descriptive statistics computed by the authors utilizing primary empirical survey data (*N* = 218). The table presents the mean values and corresponding standard errors (SE) for four evaluative dimensions across three distinct operational classifications of the Chinese Hot Spring MLR Model. All evaluative dimensions were quantitatively assessed using a standard 5-point Likert scale.

#### 3.5.2 Comparative analysis of the Chinese hot spring MLR model variants across dimensions

Post hoc comparisons using Kruskal–Wallis tests with Mann–Whitney U pairwise analyses (corrected for Bonferroni) revealed significant differences across Chinese Hot Spring MLR Model variants in professional quality, comfort level, and pricing setting, excluding service attitude (Table 10).

**Table 10:** Comparison of three types of the Chinese Hot spring MLR models using the kruskal–wallis test and *post hoc* Mann–Whitney U tests with Bonferroni correction.

Dimensions	Statistical comparisons between dimensions (*α*= 0.05)				
	H (**df**, n)	*P*-value	a posteriori test	U_(2)_	*P*-value
Medical Quality	17.424 (2,495)	<0.001***	A≠B	10,020.0	<0.001***
			B≠C	18,760.5	<0.01**
Service Attitude	4,043 (2,495)	n.s.			
Comfort Level	15.447 (2,495)	<0.001***	A≠B	11,571.0	<0.05*
			B≠C	20,012.0	<0.001***
Price Setting	18.835 (2,495)	<0.001***	A≠B	9,983.5	<0.001***
			A≠C	8,205.0	<0.001***

Computed by the authors utilizing primary empirical survey data. Derived from the overarching study cohort (N=218), a total of n=495 valid evaluative observations were analyzed across the three operational typologies. The table presents the nonparametric Kruskal–Wallis H statistic to evaluate distributional differences among the models. Significant main effects were subsequently analyzed via a posteriori Mann–Whitney U tests, employing the strict Bonferroni correction to adjust for multiple comparisons. Typology designations: A = Western Medicine Hot Spring Medical and Health Care Model; B = Traditional Chinese Medicine Hot Spring Medical and Health Care Model; C = Non-Medical Hot Spring Health and Wellness Model. df = degrees of freedom; n.s.=not significant. Statistical significance is denoted as follows: * P<0.05, ** P<0.01, *** P<0.001.

## 4 Discussion

### 4.1 Comprehensive analysis of contemporary health issues in China

Amid global population aging and high non-communicable disease prevalence, public healthcare expectations now transcend curative interventions (22–24). The medical community increasingly recognizes suboptimal health—a borderline state between wellness and disease—as a critical public health priority (25).

### 4.2 Analysis of target population characteristics in the Chinese hot spring MLR model

The U.S. Centers for Disease Control and Prevention (CDC) defines suboptimal health with persistent and prominent symptoms as Chronic Fatigue Syndrome (CFS) (26)—a complex, disabling condition marked by unexplained, recurrent, or continuous fatigue lasting more than six months. Patients often experience a range of somatic and neuropsychiatric symptoms, including musculoskeletal pain, headaches, impaired concentration, poor sleep quality, and emotional distress. These symptoms substantially impair daily functioning, limiting individuals' capacity to work, study, and maintain social engagement (27, 28).

According to the 2023 World Health Statistics Report, chronic diseases accounted for nearly 41 million deaths globally in 2019, representing almost three-quarters of all deaths and now ranking as the primary contributor to the global disease burden (29). In China, the growing aging population and rapid lifestyle transitions have led to a rising incidence of chronic conditions, increasingly affecting younger populations. Over 190 million older adults live with at least one chronic disease. Among adults aged 18 and above, hypertension affects 25% of the population, while dyslipidemia affects approximately 40%. Chronic diseases now contribute to more than 80% of all deaths and account for over 70% of the total national disease burden (30).

As a complementary approach to conventional medicine, spa therapy using thermal spring water exerts its therapeutic effects through an interplay of physical mechanisms, chemical constituents, and psychological influences. The physical properties of thermal water involve both thermal and mechanical stimuli. Heat exposure promotes metabolic activity, elevates catecholamine and renin levels, and enhances cardiac output—effects that have been shown to relieve suboptimal health conditions, such as chronic fatigue (31, 32). Thermal stress also induces the expression of heat shock proteins (HSPs), which strengthen cellular resistance to physiological stress (33). Mechanical stimulation, including hydrostatic pressure, activates cutaneous mechanoreceptors and modulates nociceptive pathways via the spinothalamic tract. This neural modulation reduces pain transmission and may slow the progression of chronic inflammation, which is commonly associated with long-term illnesses.

The chemical composition of thermal spring water not only guides classification but also determines therapeutic specificity. Carbon dioxide (34) and sulfide-rich (35, 36) waters help manage chronic cardiovascular conditions, such as hypertension. Bicarbonate and alum-containing springs benefit individuals with chronic dermatological disorders (37), while silicate springs form a protective film on the skin, supporting epidermal barrier function and facilitating scar repair (38).

Beyond physical and chemical effects, spa therapy also demonstrates significant psychological benefits (39). Numerous studies have reported reductions in anxiety and depression following spa interventions. A meta-analysis of 17 randomized controlled trials found a moderate overall effect, with the greatest improvement observed in anxiety symptoms (13). Previous research has established associations between psychological symptoms (PS) and somatic health status (SHS) (40, 41), particularly concerning anxiety (42, 43). A recent network analysis confirmed a direct link between SHS and anxiety-related symptoms (25). In older adult Chinese populations with comorbid depression and anxiety, interventions targeting core anxiety symptoms proved especially effective. Addressing bridging symptoms, such as poor sleep, also contributed to the simultaneous relief of both anxiety and depression (44).

In summary, spa therapy offers a targeted and evidence-based intervention for psychological conditions such as anxiety, aligning with the principles of the Chinese Hot Spring MLR Model. It also provides supportive therapeutic benefits for chronic non-communicable diseases affecting the skin (45), musculoskeletal system (46, 47), cardiovascular system (48), and metabolism (49, 50).

This study identifies a distinct population distribution for the Chinese Hot Spring MLR model, reflecting a “prevention-centered, rehabilitation-supported” framework. Sub-healthy individuals comprise the largest segment (44.50%), representing the primary focus for risk prevention and proactive health management. Those engaged in rehabilitation—including patients recovering from illness and individuals with chronic conditions—account for 28.90%, forming a secondary layer of therapeutic demand. The older adult and disabled population represents 26.61%, indicating a group with long-term care needs.

Furthermore, a critical comparative synthesis of our findings with the broader international literature elucidates substantial paradigm divergences. While established studies on traditional European balneotherapy predominantly highlight its disease-modifying efficacies in specific rheumatological or dermatological cohorts (51), our empirical data indicate a profound structural shift within the Chinese context. Specifically, the target demographic in this study disproportionately prioritizes the macroscopic integration of “long-term care” (26.61%) and “preventive sub-health management” (44.50%) over isolated clinical symptom relief. We interpret this pronounced cross-cultural discrepancy not merely as a variance in consumer preference, but as a direct reflection of China's unique sociodemographic pressures—most notably, an accelerated aging trajectory and the resultant systemic demand for combined medical-nursing modalities. Consequently, unlike the highly specialized clinical orientation characteristic of Western medical spa frameworks, the Chinese Hot Spring MLR Model operates as an integrated public health buffer rather than a singular therapeutic intervention.

From an epidemiological perspective, the characteristics of the target population closely align with common health risks in modern society. The primary demographic consists of individuals aged 26 to 45 years (77.52%) and is predominantly urban (76.15%), indicating a strong trend towards urbanization. This population frequently experiences high occupational stress, prolonged sedentary behavior, insufficient sleep, and psychological strain—factors that contribute to elevated risk for sub-health states and chronic non-communicable diseases (52, 53).

### 4.3 Trends and influencing factors of consumer behavior in the Chinese hot spring MLR model

The traditional Chinese medicine (TCM) type represents the most prevalent form of participation within the Chinese Hot Spring MLR Model. From the practice of “hot spring bathing” documented in the Huangdi Neijing to contemporary medical hydrotherapy, China has maintained a continuous tradition of utilizing therapeutic hot springs. Within the TCM framework, medicinal hot spring baths remain a mainstream modality, frequently employed to manage musculoskeletal, dermatological, and cardiovascular conditions (54, 55). A growing body of evidence supports the clinical efficacy of these treatments, reinforcing the cultural acceptance and widespread adoption of the TCM-based Chinese Hot Spring MLR Model.

Target population surveys reveal that 46.01% of users spend less than 400 yuan per visit, while 47.91% fall within the 400–1,000 yuan range. These spending patterns reflect a market primarily composed of basic and mid-to-high-tier consumers, with services focused on general bathing, hot spring physiotherapy, and short-term wellness interventions. In contrast, only 6.08% of consumers invest more than 1,000 yuan per visit—indicating limited engagement with premium medical-grade hot spring services and revealing a substantial gap in demand for professional, health-focused offerings.

The consumption level of the Chinese Hot Spring MLR Model demonstrates significant associations with several key factors, including age, educational attainment, consumption motivation, and exposure to social media promotion. Individuals aged 26 to 55 display significantly higher engagement compared to other age groups, with those between 36 and 55 forming the largest consumer segment (56). This trend reflects both stronger financial capacity and clearly defined health demands. Studies estimate that 48% to 59% of middle-aged individuals are in a sub-optimal state of health (57), indicating a strong inclination toward health-related spending and quality-oriented consumption.

The target population aged 56 to 65 demonstrates a marked demand for the premium Chinese Hot Spring MLR Model, driven by pressing needs for specialized, clinically grounded interventions. These findings align with previous observations from both China's forest health initiatives (58) and Slovakia's “silver economy” model (59), which targets aging populations through integrated wellness frameworks.

The relationship between educational attainment and the Chinese Hot Spring MLR Model consumption is nonlinear. Individuals with a high school education tend to spend more within the mid-to-high-end wellness segment, favoring practical and outcome-oriented services such as thermal therapy and short-term wellness interventions. In contrast, individuals with lower educational levels often exhibit limited awareness of wellness concepts and tend to rely more on traditional care approaches (60). Doctoral degree holders, on the other hand, report significantly lower spending in the basic wellness segment, likely reflecting a preference for scientifically validated, high-efficacy treatments over general wellness products (61).

Sub-health and chronic disease populations exhibit reduced spending across both the basic and mid-to-high-end consumption tiers. This trend likely stems from constrained financial capacity and long-term healthcare demands. Sub-health individuals typically remain in a preventive care phase, whereas patients with chronic conditions face ongoing medical expenses (62), which limits discretionary spending and shortens wellness durations. These groups tend to prioritize bare hot spring bathing or short-duration programs over high-cost wellness services.

Disabled individuals also demonstrate significantly reduced spending in the basic wellness segment, while those undergoing rehabilitation show decreased engagement in the mid-to-high-end tier. Economic constraints are closely linked to these patterns. Individuals with disabilities often focus on essential caregiving services and rely on public assistance or health insurance programs. Rehabilitative consumers prioritize therapeutic interventions but remain sensitive to cost, frequently opting for targeted yet affordable wellness options. The low participation among disabled groups may also reflect dependence on limited financial aid (63).

Regarding marketing channels, our empirical regression analysis identified a significant negative association between social media exposure and consumption levels within the Hot Spring MLR Model. Exploring the underlying mechanisms, established literature indicates that digital information overload frequently precipitates decision fatigue, particularly among cohorts with constrained health literacy (64). Concurrently, international epidemiological surveys—such as a recent Swedish cohort study (65)—report that younger demographics, who constitute the primary user base of social media, consistently exhibit high financial vulnerability and structurally restrict health-related discretionary spending. Synthesizing our empirical data with these documented facts, we interpret the observed negative correlation not as an ineffective marketing channel, but as a structural misalignment between the limited purchasing power of the social media demographic and the premium pricing tiers of professional medical spa services. Furthermore, acknowledging the often inconclusive nature of consumer behavior literature in this niche healthcare sector (66), a critical alternative interpretation warrants equal consideration. This negative association might also stem from a fundamental deficit of “medical trust” inherent in digital marketing (67). High-tier consumers actively seeking professional rehabilitation and long-term care may inherently perceive mass-market social media advertisements as lacking clinical credibility, preferring instead to rely on institutional medical recommendations or physician referrals (68). Therefore, this trend could equally reflect a profound psychological dichotomy where premium, evidence-based health services and social media promotion are viewed by the target demographic as mutually exclusive.

### 4.4 Demand–service alignment and optimization of the Chinese hot spring MLR model: AN IPA-based analysis

This study employs the Importance–Performance Analysis (IPA) method to assess how the target population of the Chinese Hot Spring MLR Model values the significance of various service dimensions in relation to actual service performance. The analysis yields several key insights.

First, each sub-dimension of service shows a significant positive correlation between perceived importance and evaluated performance. Among these, service attitude emerges as the most valued dimension by the target population, while professional quality receives the highest performance ratings. In contrast, transportation convenience ranks lowest in both perceived importance and actual performance.

Second, the dimensions of affordable price and transportation convenience reveal notable discrepancies between perceived importance and performance. Affordable price is considered highly important by the target population, but it often fails to meet expectations, suggesting that it is a critical pain point within the current service structure. Conversely, although transportation convenience is assigned relatively low importance, it performs well across evaluations, indicating that it remains a consistent competitive advantage within the Chinese Hot Spring MLR Model industry.

Within the IPA analysis framework, Quadrant I (“Keep Up the Good Work”) represents service attributes that score high in both perceived importance and actual performance—often considered critical success factors that warrant continued investment to maintain competitive advantage (69). However, the analysis reveals that none of the evaluated indicators in the Chinese Hot Spring MLR Model occupy this quadrant.

This finding underscores two fundamental issues. First, the model has yet to demonstrate clear strengths in any individual service dimension. Second, significant structural imbalances exist between the importance placed on service attributes and their corresponding performance. The absence of any indicators in this optimal quadrant suggests that the Chinese Hot Spring MLR Model remains in an early developmental phase, lacking a fully matured implementation system and still undergoing active exploration and optimization.

The dimensions of business model and service attitude fall into Quadrant II (“Concentrate Here”), indicating a pressing need for improvement in these areas due to their high perceived importance but suboptimal performance (69). A study on the spatial distribution of hot springs in China highlights that the inherent similarities between hot spring resources across regions contribute to significant homogenization in the development of the Chinese Hot Spring MLR Model (70). This, in turn, diminishes the uniqueness and regional distinctiveness of the Chinese Hot Spring MLR Model.

Additionally, research by Wen Yuhua and colleagues underscores that “service attitude and efficiency” are central concerns for the target population of the Chinese Hot Spring MLR Model. Given the experiential nature of these services, high standards in service quality and customer satisfaction are essential. Consequently, substantial improvements are necessary to meet consumer expectations and enhance the overall service experience (71).

Affordable price is positioned in Quadrant III (“Low Priority”), indicating that this dimension is perceived as relatively unimportant and underperforming, yet it does not warrant immediate improvement. Within the Chinese Hot Spring MLR Model, the target population consistently assigns low value to both the importance and performance of affordability. This trend reflects the characteristics of the primary consumer group—individuals actively seeking solutions to real health concerns—whose purchasing decisions are primarily influenced by therapeutic effectiveness rather than price sensitivity.

Crucially, a critical cross-study comparison of these IPA outcomes reveals a stark paradigm divergence from established international medical tourism literature. In conventional Western clinical spa and medical tourism evaluations (72), dimensions such as “professional medical quality” and “clinical efficacy” consistently emerge in Quadrant I (High Importance/High Performance), serving as the primary drivers of consumer selection. Conversely, our empirical matrix relegates “professional quality,” alongside “hot spring culture” and “transportation convenience,” to Quadrant IV (Possible Overkill)—indicating robust service delivery but disproportionately low perceived importance by the target demographic (20). We interpret this paradoxical finding not merely as an issue of resource misallocation, but as a structural manifestation of a profound cognitive misalignment within the Chinese market. Specifically, current consumers still predominantly frame the Hot Spring MLR Model through a “leisure and symptomatic relief” lens rather than acknowledging it as a systematic, evidence-based medical intervention. This critical inter-study divergence demonstrates that directly transplanting highly-medicalized Western spa standards into the current Chinese context, without simultaneously elevating public health literacy, inevitably results in systemic resource overkill and expectation mismatches.

Furthermore, inter-group comparisons across various dimensions of the different Chinese Hot Spring MLR Models reveal that the Western medicine type significantly outperforms the non-intervention type in terms of professional quality and comfort level. It also demonstrates superior performance compared to traditional Chinese medicine.

### 4.5 Limitations of the existing literature

Despite the growing body of literature on balneotherapy and wellness tourism, several critical limitations within the existing research must be acknowledged. First, the majority of current studies exhibit a pronounced micro-level clinical bias. They predominantly focus on the physiological mechanisms or isolated symptomatic relief of hot spring interventions for specific dermatological or rheumatological conditions (73), thereby lacking macro-level, policy-oriented evaluations regarding the systemic integration of medical, rehabilitation, and long-term care resources. Second, the existing literature largely relies on descriptive case studies or Western medical tourism frameworks (74). These paradigms inadequately capture the unique sociodemographic pressures of the Chinese context, particularly the accelerated population aging and the high prevalence of sub-health conditions. Furthermore, there is a conspicuous deficit of quantitative, consumer-centric methodologies in current health economics research concerning this topic. Previous studies rarely employ multidimensional analytical frameworks, such as the Importance-Performance Analysis (IPA), to systematically map the behavioral determinants and supply-demand discrepancies within the target population, as these models are conventionally restricted to the commercial hospitality sector rather than integrated healthcare systems (75). Consequently, the existing literature frequently fails to provide a rigorous, data-driven foundation for optimizing service configurations—a critical empirical gap that the present study actively addresses.

## 5 Conclusion

Drawing upon primary empirical data (N=218), this study conceptually synthesizes evidence-based insights regarding the Chinese Hot Spring MLR Model. The quantitative analysis demonstrates that consumption behaviors within this integrated care pathway are significantly associated with a complex interplay of sociodemographic determinants and psychosocial motivations. Furthermore, the Importance-Performance Analysis (IPA) conceptually identifies a critical structural misalignment: current service provisions are disproportionately concentrated in peripheral amenities with relatively lower strategic priority (e.g., traditional hot spring culture and basic infrastructure), whereas core healthcare values prioritized by consumers (e.g., business model innovation and professional service attitude) remain substantially underperforming.

Transitioning from these synthesized empirical findings to policy-oriented implications, our results suggest that the industry's developmental focus may require structural optimization. Stakeholders and health policymakers could benefit from prioritizing preventive interventions and comprehensive health management tailored specifically for sub-health populations. To effectively resolve the identified supply-demand discrepancies, institutional resource allocation could be optimized by increasing investment in business model innovation and service quality enhancement, rather than continuing conventional over-investment in basic amenities.

Finally, while this study provides foundational quantitative insights, several methodological limitations highlight specific avenues for future research. Given the cross-sectional nature of the current survey, causal relationships cannot be definitively established. Future investigations are encouraged to employ longitudinal approaches—particularly prospective cohort studies—to systematically track changes in consumer behavior and long-term health outcomes within the MLR model. Furthermore, to address the constraints of relying solely on structured survey data, methodological improvements are warranted. Future research could be enriched by integrating mixed-methods designs, specifically incorporating qualitative in-depth interviews to capture the nuanced psychological mechanisms underlying consumer decision-making. Additionally, expanding the research scope through multi-center comparative trials across diverse geographic regions would significantly enhance the generalizability of these findings and further elucidate the contextual effectiveness of integrated medical spa interventions.

## Data Availability

The raw data supporting the conclusions of this article will be made available by the authors, without undue reservation.
